# Subjective and Objective Improvement in a 39-Year-Old Male Suffering From Severe Chronic Pain and Disability Using Chiropractic BioPhysics® Protocols Following Rear-Impact Motor Vehicle Crash With a 10-Month Long-Term Follow-Up

**DOI:** 10.7759/cureus.50849

**Published:** 2023-12-20

**Authors:** Paul A Oakley, Jason W Haas, Thomas Woodham, Miles Fortner, Deed E Harrison

**Affiliations:** 1 Physical Medicine and Rehabilitation, York University, Toronto, CAN; 2 Physical Medicine and Rehabilitation, CBP NonProfit, Windsor, USA; 3 Physical Medicine and Rehabilitation, Western Plains Chiropractic, Gillette, USA; 4 Physical Medicine and Rehabilitation, CBP NonProfit, Eagle, USA

**Keywords:** musculoskeletal rehabilitation, migraine, lordosis, radiculopathy, motor vehicle crash (mvc)

## Abstract

We present the case of a patient receiving structural rehabilitation following a rear-impact motor vehicle collision (MVC). Medications did not alleviate the symptoms of the crash injuries. Resolution of injury-caused pain and disability was found following postural and structural rehabilitation treatment.

A 39-year-old male was injured in a rear-impact collision between two very large vehicles. Severe migraine headaches, neck pain, and radiculopathy, as well as lower back pain, were the result of the crash. Patient-reported outcomes (PROs) demonstrated that the symptoms were causing severe disability and poor health-related quality of life (HRQoL) measures. Radiographs found spine alignment abnormalities consistent with rear impact MVC. Chiropractic Biophysics® (CBP®) structural rehabilitation was performed.

Following a treatment regimen involving strengthening weakened and damaged muscles, postural and spinal traction, postural spinal manipulative therapy (SMT), and home therapies resulted in the resolution of the symptoms. All outcome measures demonstrated improvement, including Short-Form 36 question health questionnaire (SF-36), quadruple visual analog scale (QVAS), headache disability index (HDI), neck disability index (NDI), revised Oswestry disability index (RODI), as well as significant measured improvements found on radiographs.

Spine pain and altered alignment are frequent results of MVCs. If left uncorrected, these abnormalities increase the likelihood of chronic pain and disability. Combined low back pain (LBP), neck pain (NP), headache (HA), and radiculopathy, as found in our subject, significantly pre-dispose the individual to poor HRQoL, years lived with disability (YLDs) and increased the global burden of disease (GBD). Physicians who treat injured patients should have a repeatable, reliable, valid, and efficacious method to reduce pain, increase range of motion (ROM), improve spine alignment, and improve the performance of activities of daily living (ADLs). Further, larger studies of injured patients are necessary to determine if the CBP® protocol reduces GBD caused by MVC injuries.

## Introduction

We present the case of a subject suffering since being involved in a rear-impact motor vehicle collision (MVC) involving two very large pick-up trucks. The suffering was severe and led them to seek treatment first at the hospital and later with his general practitioner. The patient's general practitioner recommended rehabilitation due to the failure of the medications. Due to non-recovery and worsening of symptoms, they sought treatment at a rehabilitation center in Gillette, WY, USA.

Headache (HA), neck pain (NP), low back pain (LBP), and radiculopathy causing disability are common consequences of rear impact MVCs, and treatment options vary from medications to physiotherapy, injections, and surgery [1-3]. Given the significant global burden of disease (GBD) due to spine pain, treatment options that are safe, economical, and effective are imperative not only to improve the patient's suffering but also to lessen the economic and societal burden of these disabling conditions [4,5].

This case represents a single report to add to a growing body of research assessing these methods. The treatment successfully lessens pain and disability, and re-assessment found significant spine alignment improvements consistent with prior studies [6-9]. Long-term 10-month follow-up evaluation found the improvements were stable, and continued improvement was found both subjectively and objectively. Chiropractic BioPhysics® (CBP®) may offer a treatment option for physicians and clinicians treating patients injured in MVCs, reducing pain and lessening GBD [6-9].

## Case presentation

A 39-year-old male (height of 175.3 and weight 98.9kg) drove a full-sized pick-up. He was stopped at an intersection when he was struck from behind by a similarly sized vehicle. The collision caught the patient unaware, and he reported being violently thrown forward against the seat belt and then violently thrown backward into the seat. The airbag did not deploy on the target vehicle; it is unknown if the airbag was deployed in the bullet vehicle. He reported the NP and HA to the scene's emergency medical services (EMS). Throughout the next few hours, his symptoms worsened, and he sought evaluation at the emergency room (ER). Following an examination and no imaging, he was diagnosed with cervical sprain/strain and was prescribed multiple medications. The medications prescribed were Norco, Ibuprofen, Lidoderm patch, Robaxin, and Tylenol. His most debilitating symptom was severe migraine HA that did not relent for five days and was diagnosed by his general practitioner, who referred him to a Gillette, Wyoming, USA facility for spine rehabilitation.

At initial rehabilitation, the assessment included history, orthopedic tests, neurological testing, balance assessment, and grip strength, measured along with visual range of motion (ROM) for pain and full-spine upright radiography. The patient reported near-constant head pain measured on the HDI (98/100), indicating severe disability [10]. Constant NP-causing disability found using the NDI (64/100) indicated severe disability [11]. The patient reported the NP radiated into the right trapezius and right arm. The radiating pain was associated with numbness and tingling and caused weakness in the right arm. The patient also reported LBP measured on the RODI (26/50), indicating severe disability [12].

QVAS for pain was assessed for cervical, thoracic, and lumbar spine pain (67/100, 37/100, and 13/100, respectively), indicating moderate to severe spine pain. Rand short-form thirty-six question (SF-36) health status questionnaire found multiple deficits compared to normative data. The questionnaire found general health perception was reduced (35/100), physical function deficit due to pain (35/100), physical ability due to (0/100), emotional deficit due to pain was normal (100/100), social deficit was severe due to pain (0/10), and mental health deficit due to pain (32/100), and energy/fatigue due to pain (15/100) [13]. Combined, these measures demonstrated a severe reduction in HRQoL due to the pain and suffering since this crash. 

Cervical compression pain provocation testing was found to be positive for local NP, and radiating radicular pain and cervical distraction testing gave the patient temporary pain reduction. Grip strength testing found a deficit on the right (90lbs/119.7lbs), while the grip on the non-radicular left side found no deficit [14]. Single leg standing balance test found 3 seconds sustained with eyes closed on the left leg and 4 seconds on the right. This is significantly reduced for the patient's age (normal 14s for 39 old) [15]. Visual ROM for pain assessment in the cervical spine found restriction and pain with flexion, extension, and right and left lateral flexion. Cervical rotation was restricted but not as painful.

**Figure 1:**
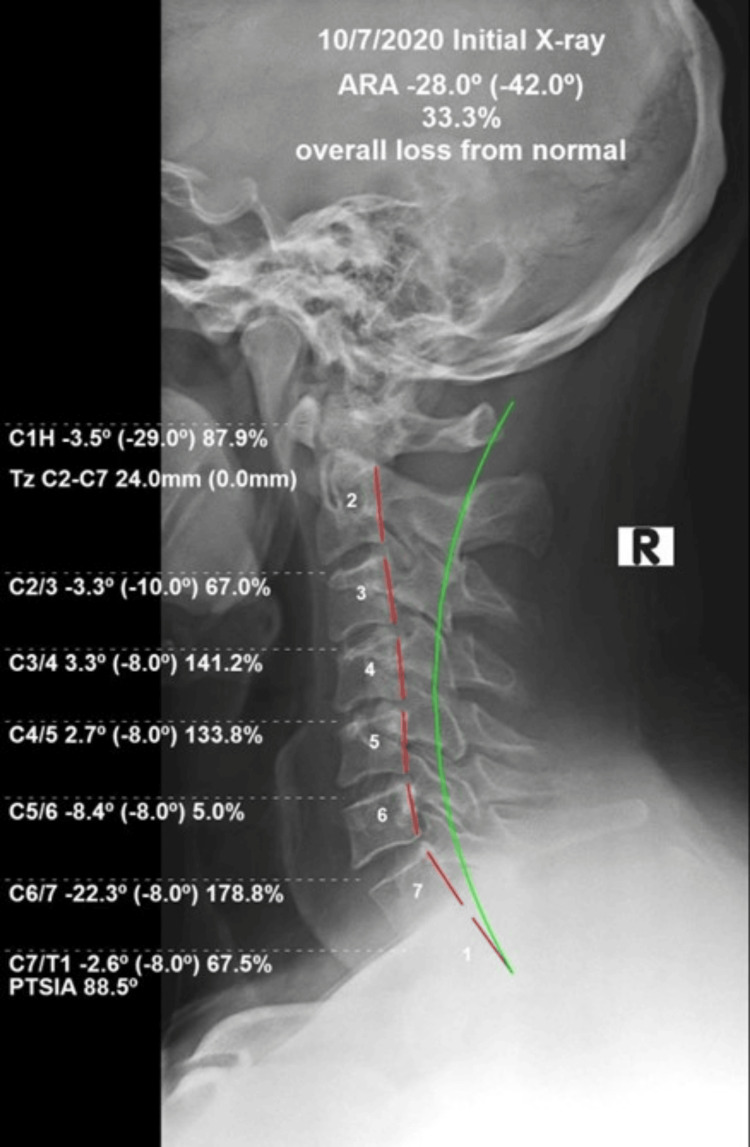
Post-MVC pre-treatment lateral cervical radiograph The red dashed line indicates the abnormal position of the vertebrae using the Harrison Posterior Tangent Method. The green line represents ideal cervical lordosis.

Radiographic full spine analysis was performed using PostureRay® (Trinity, FL, USA). This repeatable, reliable system uses machine learning intelligence to assess intersegmental relative rotation angles (RRA), as well as absolute rotation angles (ARA) of global regions [16-18]. The patient demonstrated significant loss of cervical lordosis ARA (C2-C7 -28.0°) (-42° is ideal), and intersegmental RRA demonstrated flexion kyphotic deformity C3/C4 3.3° (-8.0° is ideal), C4/C5 2.7° (-8.0° is ideal), and significant increase compared to normal extension at C6/C7 -22.8° (-8.0° is ideal). The atlas plane line (APL) was dramatically reduced and flexed, showing 87.9% loss of normal position -3.5° (-29.0° is ideal). Substantial anterior head translation (AHT) was measured (+TzH /forward translation of the head along the z-axis) at 24.0mm (0.0mm is ideal). Following the initial evaluation, the patient was found to have trauma-induced snap-through buckling consistent with MVC and pain diagnoses for cervical, thoracic, and lumbar regions. The patient was provided informed consent and initiated treatment for his abnormal spine conditions and pain (Fig 1).

CBP® therapy consists of Mirror Image® (MI) exercises designed to strengthen postural muscles and specifically used to improve the abnormal spine configuration as demonstrated on the radiographs [6]. Specific exercises to increase the cervical musculature in extension were prescribed, and the ProLordotic™ (Circular Traction LLC., Huntington Beach, CA, USA) neck exercises were used while under WBV using the Power Plate® WBV device (Power Plate USA, Northbrook IL., USA) [19]. Exercises were performed at repetitions of 5 seconds with 2 seconds rest in between for 2 minutes. Seated thoracic extension exercises were performed on the Power Plate® for 2 minutes per treatment session. MI® traction was utilized and consisted of a seated position with the head in slight extension while a distraction force was applied. A secondary force was placed in the mid-cervical spine, pulling from posterior to anterior (P-A), and the patient's ribcage was stabilized. The patient progressed through weight and time, starting at 8 minutes and working up to 20 minutes with 10 lbs in the distraction force and 25 lbs on the P-A load.

Additionally, spinal manipulative therapy (SMT) was used to reduce pain and increase ROM, and specific postural MI® SMT was used to improve postural position [20]. The patient was instructed to perform the postural exercises at home daily. He was a very compliant in-office patient and reported performing his home care exercises consistently. The patient was seen in the office three times per week for eight weeks, followed by a re-assessment of all outcome measures.

Results

Following the twenty-four treatments, the patient was given at least a twenty-four-hour rest before re-assessment to ensure accurate results. Symptomatic improvement was stark, and the patient was very satisfied with the treatment results. He reported that due to the treatment, his migraine headaches were improved by 80%, neck pain, and stiffness were improved by 80%, and radicular shoulder and right arm pain, numbness, and tingling were reported to be 70% improved. Thoracic pain was improved by 30%. Interestingly, he reported that his anxiety and irritability were 80% improved. Postural improvement found no coronal abnormalities, and only slight head extension (-RxH or extension rotation of the head around the x-axis) was visualized. No positive orthopedic tests were found. Grip strength improved dramatically to 110 lbs on the right and is now measured within normal limits (WNL). Balance improved, and the patient could now stand for 9s on the left and 13s on the right. All cervical and lumbar ROM was WNL without pain. He reported cessation of all of the OTC medications.

**Figure 2:**
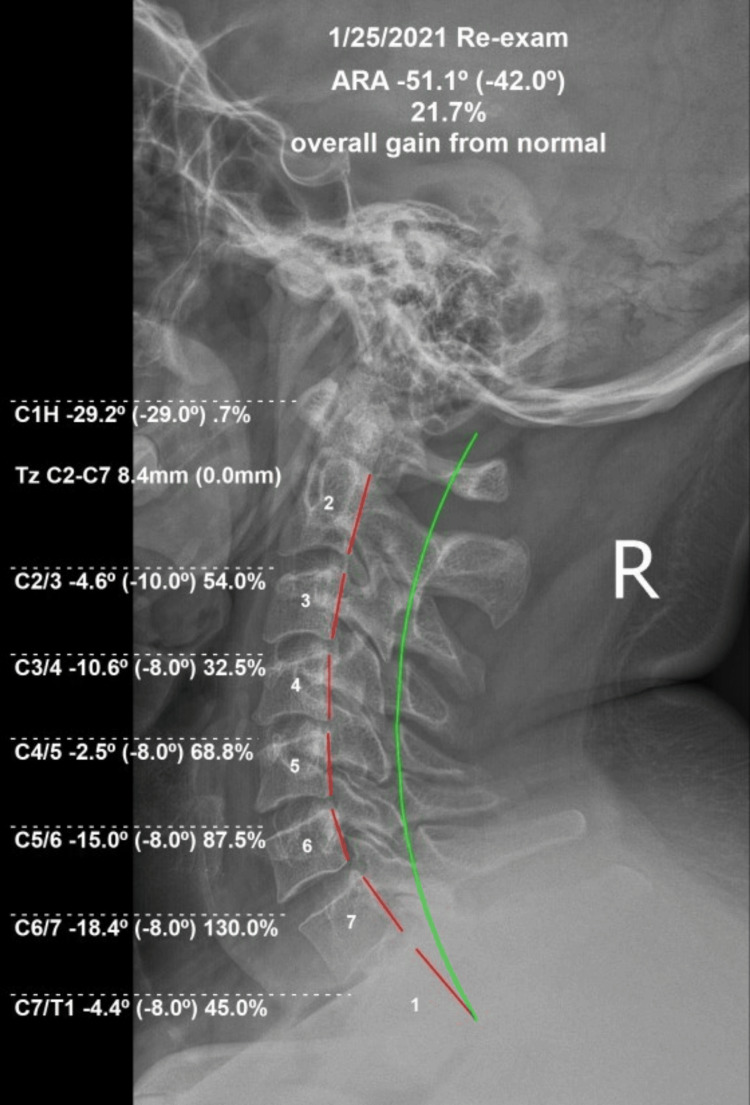
Post Treatment Lateral Cervical Radiograph The dashed red line demonstrates the restored cervical lordosis following treatment measured with the Harrison Posterior Tangent Method.  The green line represents ideal cervical lordosis.

Radiography found a significant increase in cervical lordosis from -28° to -51.1° with no kyphotic segments measured, and AHT reduced from 24mm to 8mm. APL measured as normalized (-29.2°/-29°) (Fig 2). HRQoL measures demonstrated multiple improvements across all regions. HDI improved from 98/100 initially to 24/100 at re-examination. NDI measured from 64/100 at the initial examination to 12/100. RODI improved from 26/100 to 14/100. QVAS improved in all regions correlated to symptom reduction. Neck QVAS measured 17/100 at re-exam compared to 67/100 initially, thoracic QVAS improved from 37/100 to 20/100, and lumbar QVAS measured 7/100 compared to 13/100 initially. As QVAS minimally important clinical difference (MCID) is reported as 20/100, the patient demonstrated an overall MCID for this QVAS as a significant 3.5.

SF36 was improved in all categories, with health perception (77/100), physical functioning (90/100), physical performance detriment due to pain (75/100), social function improved to 75/100, mental health (80/100), bodily pain 78/100, and energy/fatigue (65/100). This dramatic improvement in HRQoL and patient-reported outcomes (PROs) demonstrates subjective and objective improvements and continued improvement in spinal and postural alignment. The patient was instructed to continue to perform his home exercises and seek care if his symptoms return or if there is another exacerbation or injury (Table 1).

**Table 1:** SF-36 Health Status Questionnaire results

Date	Health	Physical	Physical	Emotional	Social	Mental	Bodily	Energy/
	Perception	Function			Function	Health	Pain	Fatigue
Normal	72	84	81	81	83	78	75	61
10/14/2020	35	35	0	100	0	32	23	15
1/25/2021	77	90	75	100	75	80	78	65
11/8/2021	72	95	100	100	80	68	78	60
Overall Change	35	60	100	0	80	36	55	45

At 10-month follow-up, the patient was contacted for re-assessment. He reported diligent home application of postural exercises and did not report any new injuries or exacerbations. He reported his symptoms had remained nearly resolved and continued to improve over time. He reported not being on any medications at follow-up. Headaches remained 80% improved from baseline. NP and stiffness were 90% improved from the initial evaluation; radicular pain, numbness, and tingling remained 80% improved. Thoracic spine pain was 40% improved from the initial assessment. Anxiety and irritability continued to improve and were reported to be 90% better than baseline. These are self-reported values; however, they correspond very well with the objective outcome measures recorded and presented in the table above. No postural abnormalities were found. No positive orthopedic and neurological tests were found. ROM assessment for pain was all WNL and pain-free. Grip strength assessment found normal strength at 114lbs on the right and 113lbs on the left. Single leg balance assessment with eyes closed found the patient could hold the posture for 10s on the left and 11s on the right, indicating near normal balance compared to baseline initial assessment using normative data.

**Figure 3:**
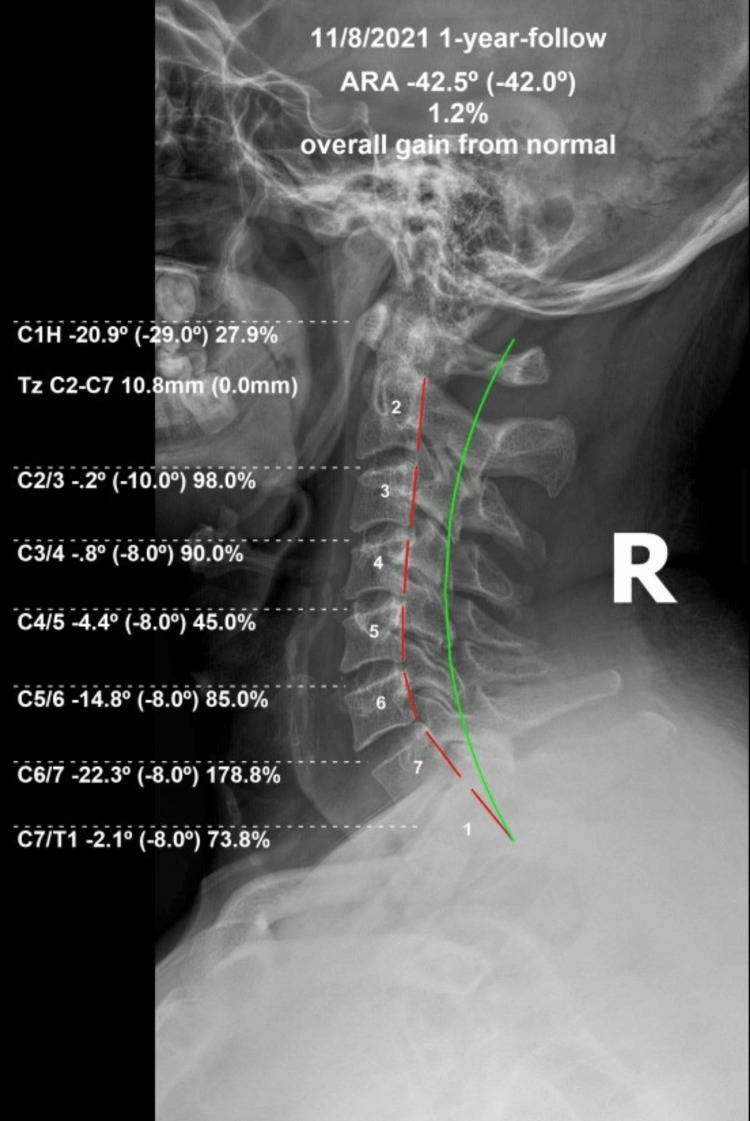
Long-term lateral cervical radiograph with stability The dashed red line is the posterior body tangents showing the lordosis maintained at follow-up.  The green line represents the ideal cervical lordosis.

 Upright spine radiography found the lordosis was still well maintained (C2-C7 -42.5°/42.0°). APL moved slightly back toward baseline (-20.9°/29.0°). No kyphotic segments were observed (Fig 3). HDI showed continued improvement (12/100), as did NDI (12/100). Cervical thoracic and lumbar QVAS were all found to be very mild (10/100). RODI showed continued improvement (12/50). All SF36 scores were at or above normal and matched normative data. The patient was very satisfied with his results long-term and was instructed to continue his home care and seek treatment if his condition worsened.

## Discussion

We present the case results of a 39-year-old male who received a significant improvement in PROs, HRQoLs, and cervical spine hypolordosis abnormality resolution. The patient demonstrated improved posture and reduced abnormal loads on the frame. Significantly, the overall cervical lordosis correction did not result in the already-existing increased extension angle at C5/C6. The patient had success initially in the CBP® program, demonstrated significant improvement at initial follow-up, and had continued reduction in pain, dysfunction, and disability at long-term follow-up. Reduced HA, NP, LBP, and radiculopathy will improve the patient's quality of life and reduce the societal burden of treatment.

NP and HA coupled with radiculopathy are common following MVCs [21-23]. LBP, MBP, and chronic pain with radiculopathies following trauma are also common outcomes of MVCs [24-26]. Resolution of poor PROs, disability scores, and objective measures rapidly following the initial trauma is a desirable clinical outcome [2]. The prolonged presence of pain beyond the initial phase of the injury will increase the likelihood that the pain will become sub-acute and lead to chronic widespread pain syndromes (CWPS) and other disabling spine conditions [27]. NP alone significantly contributes to the GBD and combined injuries from MVC that cause NP [28]. Migraine HAs, radicular pain with weakness, and sleep disturbances would only complicate an already complex clinical algorithm needed to best treat the patient [29]. Literature regarding treating complex injuries with complicated co-morbidities is scant in the medical literature [30].

Rear impact MVCs have been studied extensively, and the buckling injuries sustained in these elastic collisions can vary significantly from slight sprains/strains to sudden death [2]. The mental and physical status of the occupants, the type of vehicles, mass of the vehicles versus occupants, speed, road and tire factors, weather conditions, age of the vehicle, and safety features present will determine damage both to the vehicles and passengers [31,32]. When speed and mass are high, and Newtons of force exceed tissue integrity, hydrogen bonds will break in tissues at the molecular level and cascade depending on force, mass, and acceleration. Cellular wall disruption, inflammatory exudates, and cell wall tearing will cascade to more regions. Damage to tissues, such as ligaments, discs, muscles, and vascular tissues, can lead to mild injury up to and beyond bone fracture and catastrophic spine, cord, and nerve injury [2].

Multiple factors will determine if there will be injury to the occupants. Age, sex, body shape and size, muscle strength, nutritional and metabolic status, as well as the health of the occupants at the time of injury. These factors, coupled with the in-the-moment health of the occupants, will all play a role in the collision's short- and long-term outcomes for the injured patients [32-34].

Injuries that lead to chronic and migraine HAs, NP, and radicular symptoms, especially LBP, will increase patient GBD and further increase disability due to pain and suffering and increase years lived with disability (YLDs) [35]. Unfortunately, patients who fail OTC medication therapy or prescription NSAIDs may end up becoming reliant on opioids, steroids, and other greater-risk medications. Failure of OTC medications, NSAIDs, prescription drugs, and physical or manual therapies further increases the likelihood of more invasive interventions [2].

Beyond conservative care, further invasive procedures may attempt to alleviate suffering. Radiofrequency nerve ablation (RFA), injection of analgesic and steroid medications, discectomy, anterior and posterior screw fixation, as well as disc replacement surgeries have their success and complications [36-39]. Wrong level/wrong site surgeries, surgical risk of local infection, and sepsis can be adverse consequences. The necessity for multiple interventions and revisions if the initial surgery is not successful in improving pain and disability further worsens the prognosis [40,41]. Each progressive intervention adds further to the GBD and worsens the long-term disability outlook for the individual.

Previous biomechanical and mathematical modeling studies have demonstrated buckling injuries sustained in the cervical spine following rear-impact collisions. These collisions account for upwards of 30% of all crashes in the United States (US) and constitute the most likely cause of significant injury in MVCs [2]. Decades of studies have shown that an "s-shaped" cervical spine configuration is the most likely result for the individual in the "target" vehicle in a rear-impact collision [42-45]. The resting shape of the spine following the snap-through buckling injury places abnormal stresses and strains on the tissues, increases the production of inflammatory exudates, accelerates fibrosis, and increases vascularity to normally avascular tissues [46]. The longer the s-shaped configuration remains following trauma, the more likely imaging will demonstrate osteophyte production, disc height loss, ossification of annular fibers of the disc, fatty-infiltration of intervertebral and paraspinal musculature, as well as a greater likelihood of severe injury with any subsequent trauma [47].

Pain hypersensitivity in the damaged tissues, as well as scleratogenous referred pain, is very frequent following these injuries, and referred symptoms of numbness, tingling, shooting, and aching pain from the neck to the arm with weakness are common and commonly debilitating [48-50]. NP, migraines, and cervical radicular pain in the upper extremity are frequently associated with sleep disruption, similar to the patient in our case report. Sleep disturbances from musculoskeletal pain have many negative consequences for tissue healing, mental health, and physical performance and can increase the risk of further accidents and injury [51]. Mental health complications will rapidly worsen prognosis and further accelerate a patient toward disability because of the injury. Anxiety, especially while being in a vehicle but also in general, can commonly occur following MVC injuries, worsen prognosis, and expand possible treatment avenues if not resolved [52]. 

Being the target vehicle increased the likelihood of injury for our patient, and thus, he suffered from multiple debilitating conditions following the high mass collision of two very full-size pick-up trucks. These conditions were successfully treated with a repeatable, reliable structural and postural rehabilitation program. This protocol has a multi-modal approach to improve the function and structure of postural and deep paraspinal and extra-spinal musculature and improve the ligamentous and osseous structural position with specific traction forces and postural SMT to lessen abnormal tissue loads. Treatment involves several tissues, regions, and structures simultaneously. Gentle postural SMT is designed to reduce pain and increase ROM, and ice therapy and instructions are designed to make activities of daily living (ADLs) easier to perform and lessen dysfunction and disability. Our patient was compliant, and the program of care was re-assessed to determine progress following 24 treatments. Re-assessment found improvement in grip strength, PROs, and HRQoLs. The patient was very satisfied with care, was able to resume activities lost due to the crash, and continued to perform home exercise, traction, and postural and ergonomic changes in his daily life.

The treatment protocol used MI® exercise to improve damaged and weakened tissues. The repetitions begin small and work up to patient tolerance (10-50+). The exercises are prescribed based on PostureRay® radiographic abnormal measurements and PostureScreen Mobile® [53,54] evaluated postural photographs. Prescription MI® traction in the mirror image of the abnormal spine configuration begins very gently and in consideration of tolerance and progresses while maintaining the patient in a position that increases lordosis and lessens abnormal loading. The prolonged traction deforms ligaments with the goal of improved structure and function. Patient progress is assessed at every treatment with subjective symptom reportage and a numerical rating scale for pain (NRS) or VAS.

Following the first re-assessment, the patient resumed normal ADLs without interference; he continued his exercises and was re-assessed nearly a year later; he continues to experience increased reduction in pain and dysfunction. Significantly reducing his likelihood of future disability. He demonstrated through HRQoL measures that he continued to improve and his future likelihood of disability due to pain and suffering from the injuries sustained in this MVC.

The improvements in this patient add to the growing body of evidence that demonstrates conservative therapies provide short- and long-term improvements in patients suffering from migraine HAs, NP, radiculopathy, and numbness, as well as disability and dysfunction due to LBP. Safe, economical, repeatable, reliable, and efficacious therapies for patients injured in MVCs are necessary given the millions of rear-impact collisions in the US alone yearly and the hundreds of millions of humans injured yearly in MVCs globally [55].

Spinal structural rehabilitation treatment protocols have been verified across many investigations ranging from randomized clinical trials (RCTs) to cohort studies, and straightforward single-subject case reports such as this study. Statistical analysis has demonstrated the repeatability in the restoration of cervical lordosis, and improvements in PROs from RCTs using CBP methods are matched in the cohort studies and case reports, which is confirmed in the systematic reviews (SRs) [56-60]. Future meta-analyses and larger clinical trials are necessary and should be funded to confirm the benefits of structural rehabilitation on injured patients. Conservative methods could provide physicians and therapists who treat HAs, NP, LBP, and injured patients with economical and reproducible therapy for a tremendous portion of the global population. Limitations of this study are the single subject involved, the use of multi-modal therapeutic programs, and the lack of longer-term (5 year+) outcome measures assessing sustained improvement in subjective and objective outcomes following spine and postural structural rehabilitation.

## Conclusions

This case report documents the successful subjective, objective, and radiographic improvements in a car crash-injured patient using conservative spinal rehabilitation protocols with long-term follow-up. Single-subject case reports may seem trivial, but they are crucial in understanding the foundations of intervening with any patient's conditions and the individual patient needs that must be met at that time. 

Multi-modal applications may be a limitation as they can increase the research "noise" because it may be harder to understand which of the MI® interventions caused the subjective and objective improvements. However, it may be that the multi-modal approach in total is responsible for improving symptomatology. Larger RCTs may provide insight into certain aspects of a treatment approach, but case studies provide clinicians, therapists, and physicians with a real-world understanding of the possibilities of their treatment for that individual patient. This case demonstrates rapid improvement in an MVC-injured patient. Larger and repeated studies may confirm if CBP® reduces GBD.
